# Hypertriglyceridemia is associated with stroke after non-cardiac, non-neurological surgery in the older patients: A nested case-control study

**DOI:** 10.3389/fnagi.2022.935934

**Published:** 2022-11-14

**Authors:** Chaojin Chen, Qianyu Wen, Chuzhou Ma, Xiaoyue Li, Tengchao Huang, Jie Ke, Chulian Gong, Ziqing Hei

**Affiliations:** ^1^Department of Anesthesiology, The Third Affiliated Hospital of Sun Yat-sen University, Guangzhou, Guangdong, China; ^2^Big Data and Artificial Intelligence Center, The Third Affiliated Hospital of Sun Yat-sen University, Guangzhou, Guangdong, China; ^3^Department of Anesthesiology, Shantou Central Hospital, Shantou, Guangdong, China; ^4^Department of Neurosurgery, Third Affiliated Hospital of Sun Yat-sen University, Guangzhou, Guangdong, China; ^5^Guangzhou AID Cloud Technology Co., Ltd, Guangzhou, Guangdong, China

**Keywords:** elderly patients, hypertriglyceridemia, postoperative stroke, non-cardiac non-neurological surgery, sensitivity analysis

## Abstract

**Introduction:**

Geriatric postoperative stroke is a rare but serious complication after surgery. The association between hypertriglyceridemia and postoperative stroke remains controversial, especially in older patients undergoing non-cardiac, non-neurological surgery. The study aims to address this clinical dilemma.

**Materials and methods:**

We conducted a nested case-control study among 9601 aged patients undergoing non-cardiac non-neurological surgery from October 2015 to 2021. A total of 22 positive cases were matched for the surgical type and time, to 88 control patients by a ratio of 1:4. The effect of hypertriglyceridemia on the occurrence of postoperative stroke within 30 days after surgery was estimated using conditional logistic regression analysis by adjusting to various potential confounders.

**Results:**

A total of 22 cases developed ischemia stroke after surgery, and compared with the non-stroke group, they had more postoperative ICU admission, longer postoperative hospitalization and higher total cost (all *p* < 0.05), and more patients were presenting with preoperative hypertriglyceridemia [8 (36.4%) *vs.* 15 (17.0%), *p* = 0.045]. There was a significant association between hypertriglyceridemia and postoperative stroke, with adjusted odds ratios of 6.618 (95% *CI* 1.286, 34.064) (*p* = 0.024). The above results remained robust in the sensitivity analyses.

**Conclusion:**

Among older patients undergoing non-cardiac and non-neurological surgery, hypertriglyceridemia was associated with significant increased risk of postoperative stroke.

## Introduction

Postoperative stroke is a rare but serious postoperative complication associated with high disability and fatality rates. The incidence of postoperative stroke was approximately 0.01–1% in patients undergoing non-cardiac, non-neurological procedures (Mashour et al., 2011, 2014) and even up to 9–10% in patients undergoing difficult heart surgery and neurosurgery (Vlisides and Mashour, 2016; Mrkobrada et al., 2019). However, the 30-day mortality rates in patients who experience postoperative stroke ranged from 21 to 26%, which were 8 times greater than those without postoperative stroke (Mashour et al., 2011; Wang et al., 2019; Benesch et al., 2021). Meanwhile, patients with postoperative stroke also have longer hospital stay and higher risk of being discharged to long-term care facilities (Lindberg and Flexman, 2021). Notably, perioperative covert stroke was reported to occur in up to 7% of the older patients undergoing non-cardiac, non-neurological surgery (Mrkobrada et al., 2019). As the population ages, age of patients undergoing surgery has been increasing, the geriatric stroke after surgery represents a significant public health burden worldwide and early prediction and intervention help to improve the postoperative outcomes of the older patients (Minhas et al., 2020).

Multiple studies have reported the risk factors for postoperative stroke in patients undergoing non-cardiac, non-neurological surgery, including dyslipidemia, overweight and obesity, and metabolic syndrome (Kernan et al., 2014). Whereas, to our knowledge, few study is focusing on the older populations (van Lier et al., 2009; Bahrainwala et al., 2011; Dong et al., 2017; Kwon et al., 2021). Compared with hypercholesterolemia which is tightly associated with stroke (Sacco et al., 2008), hypertriglyceridemia is another type of dyslipidemia that received lesser attention, as its role in stroke remains controversial (Kivioja et al., 2018). On one way, patients with hypertriglyceridemia were also reported to have a significantly increased risk of incident ischemic stroke (Toth et al., 2018; Wang et al., 2018). On the other way, Anxin Wang and his colleague did not observe any significant association between triglycerides variability and ischemic or hemorrhagic stroke (Wang et al., 2020). In another nested case-control study which enrolled 5475 patients with ischemic stroke, 4776 patients with intracerebral hemorrhage, and 6290 control patients, it was demonstrated that hypertriglyceridemia were weakly correlated with ischemic stroke risk (Sun et al., 2019). To our knowledge, whether preoperative hypertriglyceridemia is associated with postoperative stroke in the older patients receiving non-cardiac, non-neurological procedures has not been reported.

To explore the risk factors for postoperative stroke in older patients undergoing non-cardiac, non-neurological surgery, we conducted a nested case-control study using a large retrospective cohort in our center, and found that hypertriglyceridemia is associated with stroke after non-cardiac, non-neurological surgery in the older patients, which enable early intervention to improve their postoperative outcomes.

## Materials and methods

### Study design

The current study was designed as a nested case-control study in a large retrospective cohort of older patients undergoing non-cardiac and non-neurosurgical surgery. The local hospital ethics committee (The Third Affiliated Hospital of Sun Yat-sen University) approved the study protocol [No. (2019)02-609-02]. No formal informed permission was required because patients were not subjected to investigational actions and unidentifiable patient information was derived from electric health records (EHR).

### Study population

All patients over 65 yrs old who underwent inpatient surgical procedures in our center from April 2015 to 2021 were included. Patients with any of the following conditions were excluded: (1) patients undergoing cardiac or neurosurgical procedures, including surgeries of major arteries such as aneurisms of thoracic aorta, (2) patients with preoperative stroke or stroke occurred beyond 30 days after surgery.

### Selection of cases and controls

Postoperative stroke is defined as a brain infarction of ischemic or hemorrhagic etiology that occurs within 30 days after surgery (Leary and Varade, 2020). Positive cases were patients with a new focal neurologic impairment of cerebral origin that lasts more than 24 h, or a computed tomography [CT] hemorrhage (Ferrara, 2020). The diagnosis of stroke was made by a neurologist and an anesthesiologist who reviewed all the EHR. Notably, positive cases included diagnoses established solely on clinical grounds (i.e., without CT abnormalities). Cases (with stroke) and controls (without stroke) were matched on type and time of surgery. For every patient with postoperative stroke, four control patients were selected who underwent the same surgery during the same period (as close as possible to the surgical date of the case) but did not experience a postoperative stroke. A ratio of 1:4 is known to yield the best results taking into account the effort to collect the information of the controls (Zhou et al., 2021). When an identical type of surgery was not available, a patient who had the most similar procedure was chosen as a control.

### Data collection

Based on previous literature, the clinical data related to demographics, detailed medical histories or perioperative variables associated with postoperative stroke were collected from the EHR system according to our earlier report (Chen et al., 2022). Demographic characteristics related to older patients including age, gender, and body mass index (BMI). Preoperative comorbidity include hypertension, diabetes, cerebrovascular disorders, carotid stenosis, coronary heart disease, atrial fibrillation, renal insufficiency, systemic lupus erythematosus (SLE), chronic obstructive pulmonary disease (COPD), history of drinking and smoking, hemodialysis and intubation before surgery. Preoperative mediations include β blocker, anticoagulant and antiplatelet agents. Preoperative laboratory findings included hemoglobin (HGB), hematocrit (HCT), platelets (PLT), monocyte, neutrophil (NEUT), high sensitivity C reactive protein (hsCRP), creatinine, blood urea nitrogen (BUN), estimated glomerular filtration rate (GFR), uric acid (UA), glucose (GLU), gamma-glutamyl transpeptidase (GGT), alanine transaminase (ALT), aspartate aminotransferase (AST), total bilirubin (TBILI), indirect bilirubin (IBILI), total cholesterol (TC), high-density lipoprotein cholesterol (HDL-C), low-density lipoprotein cholesterol (LDL-C), triglycerides, thrombin time (TT), activated partial thromboplastin time (APTT), international normalized ratio (INR), and fibrinogen. Intraoperative variables included administration of dexmedetomidine, parecoxib sodium, flurbiprofen axetil, dexamethasone, metylprednisolon, furosemide and mannitol, intraoperative infusion of crystal, colloid and red blood cell (RBC), volume of blood loss and urine output, and duration of surgery. Postoperative outcomes were also collected, including intensive care unit (ICU) admission, postoperative hospitalization, total cost and in-hospital death.

### Potential confounders

As the normal range of triglycerides was 0.34–1.92 mmol/L in our hospital, the hypertriglyceridemia was defined as triglycerides concentration >1.92 mmol/L. Variables potentially confounding the association between hypertriglyceridemia and postoperative stroke were predefined based on existing literature, clinical experience and our available EHR system, including age, gender, hypertension, diabetes, concentration of fibrinogen, and duration of surgery.

### Statistical analysis

Differences in clinical characteristics between the positive cases and controls were compared using the Student’s *t*-test for continuous variables which were normally distributed, and the non-parametric Kruskall-Wallis test was used for continuous variables with non-normal distribution. Categorical variables were compared using the Pearson chi-square test or the Fisher exact test. Conditional logistic regression analysis was conducted to estimate association between hypertriglyceridemia and postoperative stroke (Arfè et al., 2016; Glanz et al., 2018). We adjusted for different number of potential confounders in the conditional logistic regression, based on the event per variable (EPV), our experience and the data integrity. The odds ratio (OR) and 95% confidence intervals (CI) were calculated, respectively (Zhang et al., 2019; Zhao et al., 2021).

Several sensitivity analyses were performed to evaluate the robustness of the association between hypertriglyceridemia and postoperative stroke. We analyzed whether the association would change if individuals who underwent liver transplantation were removed. We reanalyzed the association if individuals who underwent carotid endarterectomy were removed. Given the possible impact of anesthesia type on postoperative stroke, we evaluated the association between hypertriglyceridemia and postoperative stroke in those patients with general anesthesia. All analyses were performed using R (release 2.13.1; R Foundation for Statistical Computing, Vienna, Austria).

## Results

**FIGURE 1:**
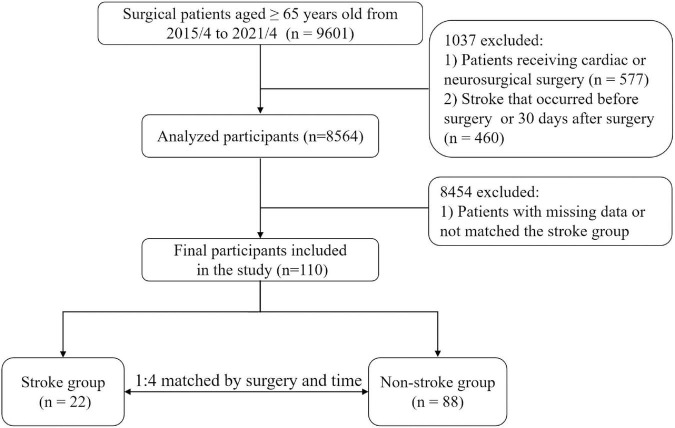
Study flowchart. The stroke diagnosis of each patient, both in the case and control groups, was re-confirmed by a neurologist and an anesthesiologist.

Of the 9601 initially screened patients in the cohort, 577 patients received cardiac or neurosurgical surgery and 460 patients had stroke before surgery or beyond 30 days after surgery were excluded. Among the 8564 patients, 22 patients (0.26%) were diagnosed as postoperative stroke within 30 days after surgery. After excluding 8454 patients with missing data or did not match the stroke group by surgery and time, a total of 22 eligible cases and 88 successfully matched controls were finally included in the study (Figure 1).

### Characteristics of the study population

In the study, we found all the 22 cases were diagnosed as ischemia stroke and all the stroke occurred within 21 days after surgery (Supplementary Table 1). Among the 110 patients (22 cases and 88 controls), the median age was 70 (67–75) years and 68 (61.8%) were men. Patients in the two groups were comparable in age [70 (67–74) *vs.* 72 (68–76), *p* = 0.564, Table 1], gender [52 (59.1%) men *vs.* 16 (72.7%) men, *p* = 0.239] and BMI [23 (21.1–25.5) *vs.* 24.8 (22.2–26.7), *p* = 0.115, Table 1].

**TABLE 1:** Preoperative characteristics of patients between two groups.

Variable	All patients	Postoperative stroke		*P* value
		
		No (*n* = 88)	Yes (*n* = 22)	
**Demographics**				
Age	70 (67;75)	70 (67;74)	72 (68;76)	0.564
Gender				0.239
Female	42 (38.2%)	36 (40.9%)	6 (27.3%)	
Male	68 (61.8%)	52 (59.1%)	16 (72.7%)	
BMI	23.4 (21.3;26)	23 (21.1;25.5)	24.8 (22.2;26.7)	0.115
**Preoperative comorbidity**				
Hypertension				0.764
No	38 (34.5%)	31 (35.2%)	7 (31.8%)	
Yes	72 (65.5%)	57 (64.8%)	15 (68.2%)	
Diabetes				0.592
No	80 (72.7%)	65 (73.9%)	15 (68.2%)	
Yes	30 (27.3%)	23 (26.1%)	7 (31.8%)	
Cerebrovascular disorders				1
Yes	0 (0)	0	0	
No	110 (100%)	88 (100%)	22 (100%)	
Carotid stenosis				1
Yes	0 (0)	0	0	
No	110 (100%)	88 (100%)	22 (100%)	
Coronary heart disease				1
Yes	0 (0)	0	0	
No	110 (100%)	88 (100%)	22 (100%)	
Atrial fibrillation				1
Yes	4 (3.6%)	3 (3.4%)	1 (4.5%)	
No	106 (96.4%)	85 (96.6%)	21 (95.5%)	
Renal insufficiency				1
Yes	0 (0)	0 (0)	0 (0)	
No	110 (100%)	88 (100%)	22 (100%)	
SLE				1
Yes	0 (0)	0 (0)	0 (0)	
No	110 (100%)	88 (100%)	22 (100%)	
COPD				1
Yes	10 (9.1%)	8 (9.1%)	2 (9.1%)	
No	100 (90.9%)	80 (90.9%)	20 (90.9%)	
Smoking				0.854
Yes	73 (84.9%)	61 (85.9%)	12 (80.0%)	
No	13 (15.1%)	10 (14.1%)	3 (20.0%)	
Drinking				0.712
Yes	78 (91.8%)	66 (93.0%)	12 (85.7%)	
No	7 (8.24%)	5 (7.04%)	2 (14.3%)	
Hemodialysis				0.2
Yes	1 (0.9%)	0 (0)	1 (4.5%)	
No	109 (99.1%)	88 (100%)	21 (95.5%)	
Intubation				0.361
Yes	2 (1.8%)	1 (1.1%)	1 (1.8%)	
No	108 (98.2%)	87 (98.9%)	21 (98.2%)	
**Preoperative medication**				
β blockers				0.326
Yes	17 (15.5%)	12 (13.6%)	5 (22.7%)	
No	93 (84.5%)	76 (86.4%)	17 (77.3%)	
Anticoagulant agents				1
Yes	0 (0)	0 (0)	0 (0)	
No	110 (100%)	88 (100%)	22 (100%)	
Antiplatelet agents				1
Yes	3 (2.7%)	3 (3.4%)	0 (0)	
No	107 (97.3%)	85 (96.6%)	22 (100%)	
**Preoperative laboratory variables**				
HGB	127 (114;135)	127.5 (114.3;135)	124 (104;135)	0.244
HCT	0.37 (0.06)	0.37 (0.06)	0.35 (0.06)	0.121
PLT	216 (79.3)	221 (81.9)	194 (65.4)	0.106
Monocyte	0.5 (0.39;0.68)	0.48 (0.39;0.655)	0.61 (0.32;0.83)	0.289
NEUT	4.25 (2.83;6.01)	4.23 (2.65;6.00)	4.40 (3.32;7.80)	0.542
hsCRP	5.15 (3.45;7.4)	5.25 (3.45;7.37)	4.56 (4.41;7.91)	0.766
Creatinine	74 (61.75;95.25)	72 (61.25;94.5)	81 (61.9;105.5)	0.342
BUN	5.62 (4.47;7.2)	5.62 (4.46;7.39)	5.605 (4.81;6.95)	0.917
Estimated GFR	75.7 (19.8)	75.9 (18.7)	75.0 (23.8)	0.894
UA	351 (108)	350 (103)	357 (127)	0.822
GLU	5.47 (4.98;6.44)	5.49 (4.90;6.30)	5.27 (5.08;6.65)	0.881
GGT	25 (16;49)	24 (16;45.25)	39 (17.75;68.25)	0.12
ALT	16 (12;24)	17 (13;23)	14.5 (10.75;28.5)	0.728
AST	21 (16;29)	21 (16;29)	20 (14;27.25)	0.766
TBILI	10.35 (7.74;15.2)	10.5 (7.8;15.2)	9.67 (7.57;15.6)	0.962
IBILI	6.6 (4.98;9.85)	6.6 (5;9.7)	6.3 (4.2;10.3)	0.84
Triglycerides	1.12 (0.8; 1.59)	1.05 (0.79; 1.54)	1.36 (0.82; 2.42)	0.103
Triglycerides categories				0.045
≤1.92	87 (79.1%)	73 (83.0%)	14 (63.6%)	
>1.92	23 (20.9%)	15 (17.0%)	8 (36.4%)	
TC	4.61 (1.13)	4.55 (1.10)	4.83 (1.21)	0.349
TC				0.367
≤5.7	92 (83.6%)	75 (85.2%)	17 (77.3%)	
>5.7	18 (16.4%)	13 (14.8%)	5 (22.7%)	
HDL-C	1.04 (0.31)	1.05 (0.30)	1.03 (0.35)	0.839
LDL-C	2.88 (0.95)	2.87 (0.96)	2.89 (0.92)	0.936
TT	13.3 (12.6;14.18)	13.3 (12.6;14.18)	13.25 (12.43;14.1)	0.914
APTT	37.3 (34.3;39.6)	37.1 (33.8;40.1)	37.85 (35.08;39.33)	0.644
INR	1 (0.94;1.1)	1 (0.94;1.11)	1.01 (0.922;1.07)	0.641
Fibrinogen	3.72 (2.94;4.66)	3.67 (3.02;4.61)	4.21 (2.75;5.28)	0.48

Data was expressed as “median (IQR)”, “mean (SD)” or “N (%)”; BMI, Body Mass Index; SLE, systemic lupus erythematosus; HGB, Hemoglobin; HCT, Hematocrit; PLT, Platelets; NEUT, neutrophil; hs-CRP, high sensitivity C-reactive protein; BUN, Blood Urea Nitrogen; GFR, Glomerular Filtration Rate; UA, Uric Acid; GLU, Glucose; GGT, Gamma-Glutamyl Transpeptidase; ALT, Alanine aminotransferase; AST, Aspartate aminotransferase; TBILI, Total bilirubin; IBILI, Indirect bilirubin; TC, Total cholesterol; HDL-C, High-density lipoprotein cholesterol; LDL-C, Low-density lipoprotein cholesterol; TT, Thrombin time; APTT, Activated partial thromboplastin time; INR, International normalized ratio.

Compared with the control group, more patients in the stroke group were presenting with hypertriglyceridemia [8 (36.4%) *vs.* 15 (17.0%), *p* = 0.045], while other preoperative variables including the comorbidities (hypertension, diabetes, cerebrovascular disorders, carotid stenosis, coronary heart disease, atrial fibrillation, renal insufficiency, SLE and COPD), administration of β blockers, anticoagulant and antiplatelet agents, other lipid indexes (TC, HDL-C, LDL-C) and other laboratory variables showed no significant differences between groups (all *p* > 0.05). The intraoperative characteristics of the 110 cases including medication, transfusion and duration of surgery were basically balanced (all *p* > 0.05, Table 2).

**TABLE 2:** Intraoperative characteristics of patients between two groups.

Variable	All patients	Postoperative stroke		*P* value
		
		No (*n* = 88)	Yes (*n* = 22)	
**Intraoperative mediation**				
Dexmedetomidine				0.762
Yes	37 (33.6%)	29 (33.0%)	8 (36.4%)	
No	73 (66.4%)	59 (67.0%)	14 (63.6%)	
Parecoxib Sodium				0.516
Yes	29 (26.4%)	22 (25.0%)	7 (31.8%)	
No	81 (73.6%)	66 (75.0%)	15 (68.2%)	
Flurbiprofen axetil				0.405
Yes	33 (30.0%)	28 (31.8%)	5 (22.7%)	
No	77 (70.0%)	60 (68.2%)	17 (77.3%)	
Dexamethasone				0.214
Yes	10 (9.1%)	10 (11.4%)	0 (0)	
No	100 (90.9%)	78 (88.6%)	22 (100%)	
Metylprednisolon				1
Yes	18 (16.4%)	14 (15.9%)	4 (18.2%)	
No	92 (83.6%)	74 (84.1%)	18 (81.8%)	
Furosemide				0.12
Yes	16 (14.5%)	10 (11.4%)	6 (27.3%)	
No	94 (85.5%)	78 (88.6%)	16 (72.7%)	
Mannitol				1
Yes	0 (0)	0 (0)	0 (0)	
No	110 (100%)	88 (100%)	22 (100%)	
**Intraoperative transfusion**				
Crystal	700 (200;1237.5)	700 (200;1200)	1000 (450;1800)	0.141
Colloid	500 (500;500)	500 (500;500)	500 (375;500)	0.22
RBC	0 (0;0)	0 (0;0)	0 (0;0)	0.457
Blood loss (ml)	50 (20;100)	50 (20;100)	50 (30;200)	0.259
Urine output (ml)	300 (100;700)	300 (100;750)	275 (100;650)	0.894
Duration of surgery	115 (66;195)	118.5 (64.75;195)	90 (67;290)	0.480

Data was expressed as “median (IQR)”, “mean (SD)” or “N (%)”; RBC, Red blood cell count.

### Prognosis of the study population

Compared with the control group, the stroke group had higher incidence of postoperative ICU admission [2 (9.1%) *vs.* 0 (0), *p* = 0.039; Table 3], longer postoperative hospitalization [19 (8, 25) *vs.* 7 (4, 12), *p* < 0.001; Table 3] and higher total cost [85060 (52524, 165378) yuan *vs.* 57586 (37592, 96905) yuan, *p* < 0.001; Table 3]. Meanwhile, the overall mortality was zero in the study.

**TABLE 3:** Outcomes of patients between two groups.

Variable	All patients	Postoperative stroke		*P* value
		
		No (*n* = 88)	Yes (*n* = 22)	
ICU admission				0.039
Yes	2 (1.8%)	0 (0)	2 (9.1%)	
No	108 (98.2%)	88 (100%)	20 (90.9%)	
Postoperative hospitalization	8 (4;16)	7 (4;12)	19 (8;25)	0.001
Total cost	60625 (380623; 100391)	57586 (37592; 96905)	85060 (52524; 165378)	0.007
In-hospital death	0 (0)	0 (0)	0 (0)	1

### Risk of stroke after non-cardiac, non-neurological surgery in the older patients

There were 8 (36.4%) and 15 (17.0%) patients had preoperative hypertriglyceridemia in the stroke and the control groups, respectively. The crude analysis showed a 4.71-fold [95% *CI* (1.13, 19.63), *p* = 0.0331] increased risk of postoperative stroke in patients with elevated triglycerides. After adjusting only for matched covariates (age, gender, fibrinogen, hypertension, diabetes and duration of surgery), we observed an increased risk of stroke in patients presenting with preoperative hypertriglyceridemia, compared with those who did not [OR 7.46, 95% *CI* (1.35, 41.22), *p* = 0.0211; Table 4]. Meanwhile, we found that after adjusting for two to eight confounders like age, gender, fibrinogen, hypertension, diabetes, duration of surgery and medications like β blockers and antiplatelet agents, preoperative hypertriglyceridemia was significantly associated with postoperative stroke (Supplementary Table 2). However, if we increased to 11 confounders and made the EPV to be 2, we found no significant increased risk of stroke between patients presenting with preoperative hypertriglyceridemia and those who did not (*P* > 0.05).

**TABLE 4:** Relationship of hypertriglyceridemia with the risk of postoperative stroke in older patients undergoing non-cardiac non-neurosurgery procedures.

	Crude		Adjusted	
		
	OR (95% CI)	*P*-value	OR (95% CI)	*P*-value
Triglycerides				
≤1.92	Ref		Ref	
>1.92	4.71 (1.13,19.63)	0.033	7.46 (1.35, 41.22)	0.021
Age(yr)	1.01 (0.93, 1.10)	0.762	1.02 (0.92, 1.12)	0.739
Gender (male)				
Female	Ref		Ref	
Male	1.90 (0.66, 5.41)	0.232	1.76 (0.57, 5.38)	0.324
Fibrinogen	1.03 (0.77, 1.37)	0.841		
Hypertension				
No	Ref		Ref	
Yes	1.29 (0.47, 3.53)	0.616	1.64 (0.51, 5.26)	0.408
Diabetes				
No	Ref		Ref	
Yes	1.33 (0.47, 3.81)	0.586	0.92 (0.29, 2.95)	0.889
Duration of surgery	1.00(0.99,1.01)	0.192		

Adjusted for age, gender, fibrinogen, hypertension, diabetes and duration of surgery at baseline.

### Sensitivity analyses

**FIGURE 2:**
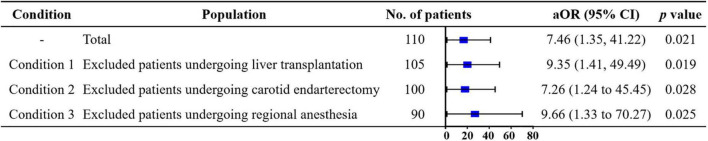
Sensitivity analysis by adjusted for certain confounders.

We conducted three sensitivity analyses to account for observed confounders (Figure 2). First, the result remained robust after excluding patients undergoing liver transplantation whose conditions and operations were much more complicated than others. Compared with patients with triglyceride ≤ 1.92, those with triglyceride > 1.92 had a higher risk of stroke [aOR 9.35, 95% CI (1.41, 49.49), *p* = 0.019]. In addition, we reanalyzed the data after excluding patients undergoing carotid endarterectomy. The risk of stroke was significantly increased in the multivariable model [aOR 7.26, 95% *CI* (1.24, 45.45), *p* = 0.028]. After excluding the patients undergoing regional anesthesia, the hypertriglyceridemia was still associated with increased risk of stroke [aOR 9.66, 95% CI (1.33, 70.27), *p* = 0.025].

## Discussion

In this nested case-control study among the older patients undergoing non-cardiac, non-neurological surgery, we found that 0.257% of the older patients suffered from a perioperative ischemia stroke, which prolonged postoperative hospital stay and increased the total hospitalization charges. Moreover, we found preoperative hypertriglyceridemia was associated with 7.46-fold increased risk of postoperative stroke after adjusting for confounding variables including age, gender, hypertension, diabetes, fibrinogen and duration of surgery.

To our knowledge, the incidence of postoperative stroke in the study was consistent with early reports in patients undergoing non-cardiac, non-neurological procedures (Mashour et al., 2011, 2014). Moreover, this was the first study to show the clinical association between hypertriglyceridemia and the probability of postoperative stroke in older patients receiving non-cardiac, non-neurological surgery. Hypertriglyceridemia is generally caused by defects in triglyceride metabolism in the older population and manifests as increased levels of plasma triglyceride (Bhatt et al., 2019). Although hypertriglyceridemia has been reported to be correlated with the increased risk of ischemic stroke according to large clinical trials (Cui and Naikoo, 2019; Gu et al., 2019), its role in stroke remains controversial and few study focuses on postoperative stroke (Glasser et al., 2016; Holmes et al., 2018). For instance, a longitudinal observational cohort study showed that in patients receiving conventional statin therapy, no significant difference was found in the incidence of non-fatal strokes between the hypertriglyceridemia group and the normal triglyceride group (HR, 1.09; 95% CI, 0.89 to 1.33; *p* = 0.42) (Nichols et al., 2018). In another population-based cohort study, researchers enrolled 961 patients with ischemic stroke and 1403 patients without ischemic stroke, and found no significant association between hypertriglyceridemia and ischemic stroke (aOR, 1.19; 95% CI, 0.94 to 1.53) (Kivioja et al., 2018). In the current nested case-control study, we included 22 older patients with postoperative stroke and matched them on type and time of surgery by a ratio of 1:4. Finally, the conditional logistic regression analysis showed that hypertriglyceridemia maintained an independent relationship with postoperative stroke in fully adjusted models, and the sensitivity analyses yielded similar results.

Triglyceride molecules are the primary means of storing and transporting fatty acids in the cells and in the plasma. Fatty acids are eliminated by oxidation within the hepatocyte or by secretion into the plasma through triglyceride-rich very low-density lipoproteins (VLDL). It is shown that with the increase of age, the activity of lipoprotein lipase and LDL receptor decrease, the levels of VLDL increase, which further increases the level of triglycerides in the plasma (Alves-Bezerra and Cohen, 2017; Johnson and Stolzing, 2019). We think hypertriglyceridemia may increase the risk of postoperative stroke by promoting atherosclerosis in the older patients (Peng et al., 2017). Firstly, hypertriglyceridemia may theoretically cause subendothelial retention of remnant particles and elicitation of endothelial dysfunction, to establish chronic inflammation in the cerevascular (Liang et al., 2022). Secondly, hypertriglyceridemia may cause hyperviscosity and promote thrombosis through a procoagulant effect involved in the disturbance of both blood coagulation and fibrinolysis, and led to postoperative stroke. Notably, the current results revealed that lipid-lowering and triglyceride reduction measures might help to prevent perioperative ischemic stroke in older patients with preoperative hypertriglyceridemia (Bhatt et al., 2019).

To further confirm the association between preoperative hypertriglyceridemia and postoperative stroke, we tried to enroll more variables potentially confounding the association between hypertriglyceridemia and postoperative stroke, include most of the demographic characteristics, preoperative comorbidity, mediations, laboratory results and intraoperative variables. However, the rule of EPV is taken into consideration in the sample size requirement of logistic regression (Peduzzi et al., 1996). Although EPV values are preferably 10 or greater, there are still several important potential confounders need to be adjusted in the study, including age, gender, hypertension, diabetes, concentration of fibrinogen and duration of surgery. As a result, the current EPV is relaxed as almost 5 samples per predictor (Vittinghoff and McCulloch, 2007). Meanwhile, the current results showed that after adjusting for 11 potentially confounding variables and relaxing the EPV to 2, no significant increased risk of stroke was found between patients presenting with preoperative hypertriglyceridemia and those who did not. The results are interesting and we think this might be attributed to the small sample size and EPV value in this condition.

### Limitations of the study

The study has several limitations. First of all, we did not collect the duration of hypertriglyceridemia in the patients and whether the triglyceride concentration level was positively correlated with the severity of stroke need to be further explored. Secondly, due to the retrospective observational design, the results lack information of preoperative and postoperative medications like lipid-lowering agents, blood pressure-lowering agents and glucose-lowering agents *etc.* In terms of agents for hypertriglyceridemia, lipid-lowering agents like omega 3 PUFA may increase bleeding events, while fibrates may increase thrombotic events. Therefore, the drug information is important to interpret the results and further studies are needed to explore and elucidate the role of preoperative and postoperative medications in the development and prognosis of postoperative stroke. Thirdly, the sample size and the positive cases were small and this limited our subgroup analysis to further explore the association between hypertriglyceridemia and postoperative stroke in different kinds of older patients, as well as the effects between different levels of hypercholesterolemia and hypertriglyceridemia in the current study. Fourthly, we did not explore whether hypertriglyceridemia is also a risk factor for other postoperative vascular events like deep vein thrombosis (DVT) and ischemic heart diseases, and further studies are needed to explore it. Finally, although we considered many confounders, the intraoperative hypotension was not correctly recorded in our database and residual confounders such as different test methods in different years might interfere with our findings.

## Conclusion

Among the older patients undergoing non-cardiac, non-neurological surgery, preoperative hypertriglyceridemia is associated with increased risk of postoperative stroke. The study findings are limited by potential confounders due to retrospective observational design and further prospective randomized clinical trials are needed to validate the finding.

## Data availability statement

The raw data supporting the conclusions of this article will be made available by the authors, without undue reservation.

## Ethics statement

The studies involving human participants were reviewed and approved by Ethics Committee of the Third Affiliated Hospital of Sun Yat-sen University. Written informed consent for participation was not required for this study in accordance with the national legislation and the institutional requirements.

## Author contributions

CC, CG, and ZH: conception and design. CC, QW, CM, XL, TH, JK, and CG: acquisition of data. CC, QW, and CM: analysis and interpretation of data. CC, QW, CM, XL, TH, JK, CG, and ZH: writing, review, and/or revision of the manuscript. CC and ZH: study supervision. All authors contributed to the article and approved the submitted version.
