# Supplementary material for: Hypertriglyceridemia is associated with stroke after non-cardiac, non-neurological surgery in the older patients: A nested case-control study
Source: Front Aging Neurosci. 2022 Nov 14;14:935934. doi: 10.3389/fnagi.2022.935934 (PMC9702075; doi:10.3389/fnagi.2022.935934)
Supplement: Supplementary file 1 [file Table_1.DOCX]

**Hypertriglyceridemia is associated with stroke after non-cardiac, non-neurological surgery in the elderly: a nested case-control study**

Chaojin Chen^1^; Qianyu Wen^2^; Chuzhou Ma^3^; Xiaoyue Li^1^; Tengchao Huang^4^; Jie Ke^5^; Chulian Gong^1#^; Ziqing Hei^1#^;

^1^Department of Anesthesiology, The Third Affiliated Hospital of Sun Yat-sen University, Guangzhou, Guangdong, China.

^2^Big Data and Artificial Intelligence Center, The Third Hospital of Sun Yat-sen University, Guangzhou, Guangdong, China.

^3^Department of Anesthesiology, Shantou Central Hospital, Shantou, Guangdong, China.

^4^Department of Neurosurgery, Third Affiliated Hospital of Sun Yat-Sen University, Guangzhou, Guangdong, China.

^5^Guangzhou AID Cloud Technology Co., LTD, Guangzhou, Guangdong, China

**Table S1. Detailed information of the 22 cases with postoperative stroke.**

| **Number** | **Type of stroke** | **Type of surgeries** | **Days after operation** |
| --- | --- | --- | --- |
| 1 | Ischemia | Orthopaedic surgery | 8 |
| 2 | Ischemia | Orthopaedic surgery | 10 |
| 3 | Ischemia | Urological surgery | 14 |
| 4 | Ischemia | Urological surgery | 2 |
| 5 | Ischemia | Gastroenterological surgery | 4 |
| 6 | Ischemia | Urological surgery | 2 |
| 7 | Ischemia | Gastroenterological surgery | 2 |
| 8 | Ischemia | Urological surgery | 3 |
| 9 | Ischemia | Orthopaedic surgery | 5 |
| 10 | Ischemia | Orthopaedic surgery | 7 |
| 11 | Ischemia | Thyroid surgery | 8 |
| 12 | Ischemia | Liver transplantation | 9 |
| 13 | Ischemia | Thoracic surgery | 20 |
| 14 | Ischemia | Thoracic surgery | 1 |
| 15 | Ischemia | Thoracic surgery | 3 |
| 16 | Ischemia | Thoracic surgery | 9 |
| 17 | Ischemia | Thoracic surgery | 10 |
| 18 | Ischemia | Orthopaedic surgery | 1 |
| 19 | Ischemia | Carotid endarterectomy. | 14 |
| 20 | Ischemia | Orthopaedic surgery | 3 |
| 21 | Ischemia | Thoracic surgery | 6 |
| 22 | Ischemia | Orthopaedic surgery | 11 |

**Table S2. Relationship of hypertriglyceridemia with the risk of postoperative stroke in older patients undergoing non-cardiac non-neurosurgery procedures.**

|  | Adjusted | |
| --- | --- | --- |
|  | OR(95% CI) | *P*-value |
| Triglycerides^1^ |  |  |
| ≤ 1.92 | Ref |  |
| > 1.92 | 4.80 (1.15, 20.10) | 0.032 |
| Age (yr) | 1.02 (0.93, 1.12) | 0.620 |
| Gender (male) |  |  |
| Female | Ref |  |
| Male | 1.98(0.67, 5.88) | 0.217 |
| Triglycerides^2^ |  |  |
| ≤ 1.92 | Ref |  |
| > 1.92 | 5.44 (1.20, 24.63) | 0.028 |
| Age (yr) | 1.02 (0.93, 1.12) | 0.700 |
| Gender (male) |  |  |
| Female | Ref |  |
| Male | 1.87(0.63, 5.53) | 0.259 |
| Hypertension |  |  |
| No | Ref |  |
| Yes | 1.63(0.51, 5.16) | 0.408 |
| Diabetes |  |  |
| No | Ref |  |
| Yes | 1.04(0.33, 3.29) | 0.942 |
| Triglycerides^3^ |  |  |
| ≤ 1.92 | Ref |  |
| > 1.92 | 7.56 (1.33, 43.05) | 0.023 |
| Age (yr) | 1.01 (0.92, 1.11) | 0.827 |
| Gender (male) |  |  |
| Female | Ref |  |
| Male | 1.71(0.56, 5.22) | 0.346 |
| Hypertension |  |  |
| No | Ref |  |
| Yes | 1.58(0.50, 5.06) | 0.437 |
| Diabetes |  |  |
| No | Ref |  |
| Yes | 0.85(0.26, 2.84) | 0.796 |
| Fibrinogen |  |  |
| Duration of surgery |  |  |
| β blockers |  |  |
| No | Ref |  |
| Yes | 1.66(0.42, 6.53) | 0.468 |
| Antiplatelet agents |  |  |
| No | Ref |  |
| Yes | 0.90(0.65, 1.26) | 0.542 |
| Triglycerides^4^ |  |  |
| ≤ 1.92 | Ref |  |
| > 1.92 | 2.74 (0.37, 20.20) | 0.327 |
| Age (yr) | 0.98 (0.92, 1.11) | 0.766 |
| Gender (male) |  |  |
| Female | Ref |  |
| Male | 1.71(0.56, 5.22) | 0.346 |
| Hypertension |  |  |
| No | Ref |  |
| Yes | 0.78(0.19, 3.20) | 0.719 |
| Diabetes |  |  |
| No | Ref |  |
| Yes | 0.97(0.19, 4.93) | 0. 986 |
| Fibrinogen |  |  |
| Duration of surgery |  |  |
| GLU |  |  |
| hemoglobin |  |  |
| TC |  |  |
| HDL |  |  |
| LDL |  |  |

^1^Adjusted for age, gender at baseline.

^2^Adjusted for age, gender, hypertension and diabetes at baseline.

^3^Adjusted for age, gender, fibrinogen, hypertension, diabetes, duration of surgery and mediations (β blockers and antiplatelet agents) at baseline.

^4^Adjusted for age, gender, fibrinogen, hypertension, diabetes, duration of surgery, GLU, hemoglobin, TC, HDL and LDL at baseline.
